# Identification of Premeiotic, Meiotic, and Postmeiotic Cells in Testicular Biopsies Without Sperm from Sertoli Cell-Only Syndrome Patients

**DOI:** 10.3390/ijms20030470

**Published:** 2019-01-22

**Authors:** Maram Abofoul-Azab, Eitan Lunenfeld, Eliahu Levitas, Atif Zeadna, Johnny S. Younis, Shalom Bar-Ami, Mahmoud Huleihel

**Affiliations:** 1 The Shraga Segal Department of Microbiology, Immunology and Genetics, Ben Gurion University, Beer Sheva 8410501, Israel; azab7808@gmail.com; 2 The Center of Advanced Research and Education in Reproduction (CARER), Faculty of Health Sciences, Beer Sheva 8410501, Israel; lunenfld@bgu.ac.il (E.Lu.); levitase@bgu.ac.il (E.Le.); atifzeadna@gmail.com (A.Z.); 3 Faculty of Health Sciences, Ben Gurion University of the Negev, Beer Sheva 8410501, Israel; 4 Fertility and IVF Unit, Department OB/GYN, Soroka Medical Center, Beer-Sheva 85025, Israel; 5 Reproductive Medicine Unit, Department OB/GYN, Poriya Medical Center, Tiberias; Azrieli Faculty of Medicine in Galilee, Bar-Ilan University, Ramat-Gan 5290002, Israel; JYounis@poria.health.gov.il (J.S.Y.); sgyss1@gmail.com (S.B.-A.)

**Keywords:** azoospermic patients, spermatogenesis, in vitro culture of spermatogonial cells, sertoli cell-only syndrome, 3-dimension culture

## Abstract

Sertoli cell-only syndrome (SCOS) affects about 26.3–57.8% of azoospermic men, with their seminiferous tubules containing only Sertoli cells. Recently, it was reported that testicular biopsies from nonobstructive azoospermic (NOA) patients contained germ cells, and that sperm could be found in the tubules of 20% of SCOS patients using testicular sperm extraction technology. Since the patients without sperm in their testicular biopsies do not have therapy to help them to father a biological child, in vitro maturation of spermatogonial stem cells (SSCs) isolated from their testis is a new approach for possible future infertility treatment. Recently, the induction of human and mice SSCs proliferation and differentiation was demonstrated using different culture systems. Our group reported the induction of spermatogonial cell proliferation and differentiation to meiotic and postmeiotic stages in mice, rhesus monkeys, and prepubertal boys with cancer using 3D agar and methylcellulose (MCS) culture systems. The aim of the study was to identify the type of spermatogenic cells present in biopsies without sperm from SCOS patients, and to examine the possibility of inducing spermatogenesis from isolated spermatogonial cells of these biopsies in vitro using 3D MCS. We used nine biopsies without sperm from SCOS patients, and the presence of spermatogenic markers was evaluated by PCR and specific immunofluorescence staining analyses. Isolated testicular cells were cultured in MCS in the presence of StemPro enriched media with different growth factors and the development of colonies/clusters was examined microscopically. We examined the presence of cells from the different stages of spermatogenesis before and after culture in MCS for 3–7 weeks. Our results indicated that these biopsies showed the presence of premeiotic markers (two to seven markers/biopsy), meiotic markers (of nine biopsies, cAMP responsive element modulator-1 (CREM-1) was detected in five, lactate dehydrogenase (LDH) in five, and BOULE in three) and postmeiotic markers (protamine was detected in six biopsies and acrosin in three). In addition, we were able to induce the development of meiotic and/or postmeiotic stages from spermatogonial cells isolated from three biopsies. Thus, our study shows for the first time the presence of meiotic and/or postmeiotic cells in biopsies without the sperm of SCOS patients. Isolated cells from some of these biopsies could be induced to meiotic and/or postmeiotic stages under in vitro culture conditions.

## 1. Introduction

About 60% of azoospermic men are diagnosed with nonobstructive azoospermia (NOA), which causes 10% of male infertility [1,2,3,4]. NOA is caused by primary, secondary, or incomplete testicular failure and the testicular histology of these patients shows hypospermatogenesis, maturation arrest, Sertoli cell-only syndrome (SCOS), or acellular seminiferous tubules [1,5,6,7].

It has been shown that seminiferous tubules from SCOS patients contain only Sertoli cells with no germ cells, and that this affects 26.3–57.8% of azoospermic men [8,9,10,11,12]. However, the presence of spermatogonial cells in testicular biopsies from nonobstructive azoospermic (NOA) patients has been demonstrated [13,14,15,16]. Despite these facts, this testicular histology may not reflect complete cases, and some SCOS patients may have some tubules with complete spermatogenesis [1,17]. It has been reported that in 20% of these patients, sperm was found in seminiferous tubules using testicular sperm extraction technology [18].

The karyotype of most SCOS men is normal, while some have smaller testis and higher levels of follicle-stimulating hormone (FSH) than normal [7,19,20,21,22,23,24,25]. Also, SCOS may be a result of Klinefelter’s syndrome, microdeletions in the Y chromosome, varicocele, chemotherapy, hypogonadotropic hypogonadism, or estrogen therapy. However, the reason for many SCOS cases is unclear [19,20,21,22,23,24,25].

To date, SCOS patients do not have therapy options to help them father a biological child [26]. In vitro maturation of spermatogonial stem cells (SSCs) extracted from the testis of azoospermic patients is one of the new suggested approaches to treat this infertility [27,28,29,30,31]. Due to the very small amount of SSCs in the testis, the very tiny size of human biopsies used, and the absence of SSC markers, this approach is very limited. In addition, the conditions for inducing SSC divisions and maturation are still obscure [27,28,29,30,31,32].

Recently, researchers have demonstrated the induction of human and mice SSC proliferation [33,34,35]. These results were achieved in mice using gelatin-coated dishes with the addition of different growth factors such as glial cell line-derived neurotrophic factor (GDNF), leukemia inhibitory factor (LIF), basic fibroblast growth factor (b-FGF), and epidermal growth factor (EGF) [2]. In 2011, Sato et al. reported the possibility of inducing fertile sperm production using a mouse testicular organ culture system [36]. In human studies, a proliferation of adult and prepubertal cancer patient SSCs could be obtained [34,35,37]. In 2009, Sadri et al. demonstrated the propagation of promyelocytic leukemia zinc finger protein (*PLZF*)-positive SSCs using StemPro rich medium and laminin-coated dishes, also with the addition of GDNF, LIF, and EGF. The propagated SSCs were xenografted in mouse testes [35]. In 2011, the propagation of human prepubertal cancer patient SSCs was achieved by culturing them in StemPro rich medium and separating them from somatic cells by passaging every several days. The cells were transplanted in busulfan-treated immunodeficient mice in order to show the efficiency of testicular biopsy preservation in young male cancer patients [34]. Also, culturing SSC-expressed *OCT4*, *Stra8*, *Piwil2*, and *VASA*, which were derived from the testes of azoospermic patients, in the presence of Sertoli cells with and without the growth factors LIF and b-FGF brought to their propagation [13]. However, another study showed the ability to propagate *GFRa1* and *a-6-Integrin*-positive SSCs derived from azoospermic patients only by the addition of GDNF, LIF, FGF, and EGF growth factors [14]. Recently, the generation of spermatid cells from NOA patients’ testicular cells using a collagen gel matrix was introduced [15]. Riboldi et al. also demonstrated the production of haploid meiotic cells that expressed synaptonemal complex protein 3 (*SCP3*) and calcium-responsive transactivator *CREST* markers from NOA patients’ CD49+ SSCs by co-culture with Sertoli cells [16]. Using an in vitro three-dimensional (3D) soft agar culture system, our group showed the differentiation of immature mouse SSCs into meiotic, postmeiotic, and even sperm-like cells [29,38,30]. Also, using a 3D methylcellulose culture system (MCS), we could develop meiotic and postmeiotic stages from premature monkey SSCs [39]. Recently, we reported the generation of meiotic, postmeiotic, and sperm-like cells in MCS from the testicular biopsies of prepubertal male cancer patients before aggressive chemotherapy [40].

In the present study, we demonstrate the presence of premeiotic, meiotic, and postmeiotic cells in biopsies without sperm from SCOS patients, and the possibility of inducing cells from some of the biopsies to meiotic and/or postmeiotic cells under in vitro culture conditions.

## 2. Results

### 2.1. Hormone Levels in Biopsies without Sperm from SCOS Patients

The hormone levels of FSH, Luteinizing hormone (LH), prolactin (Prolac), testosterone (T), and thyroid stimulating hormone (TSH) were examined in the blood of SCOS patients by radioimmunoassay. The FSH levels were higher in most of the patients compared to the normal range (Table 1). The LH levels were higher in four of the patients, and prolactin levels were in the normal range, except for two patients who showed higher levels. Testosterone levels were in the normal range (Table 1).

### 2.2. Immunofluorescence Staining of Premeiotic Markers in Testicular Biopsies from Patients with Hypospermatogenesis and SCOS

**Figure 1:**
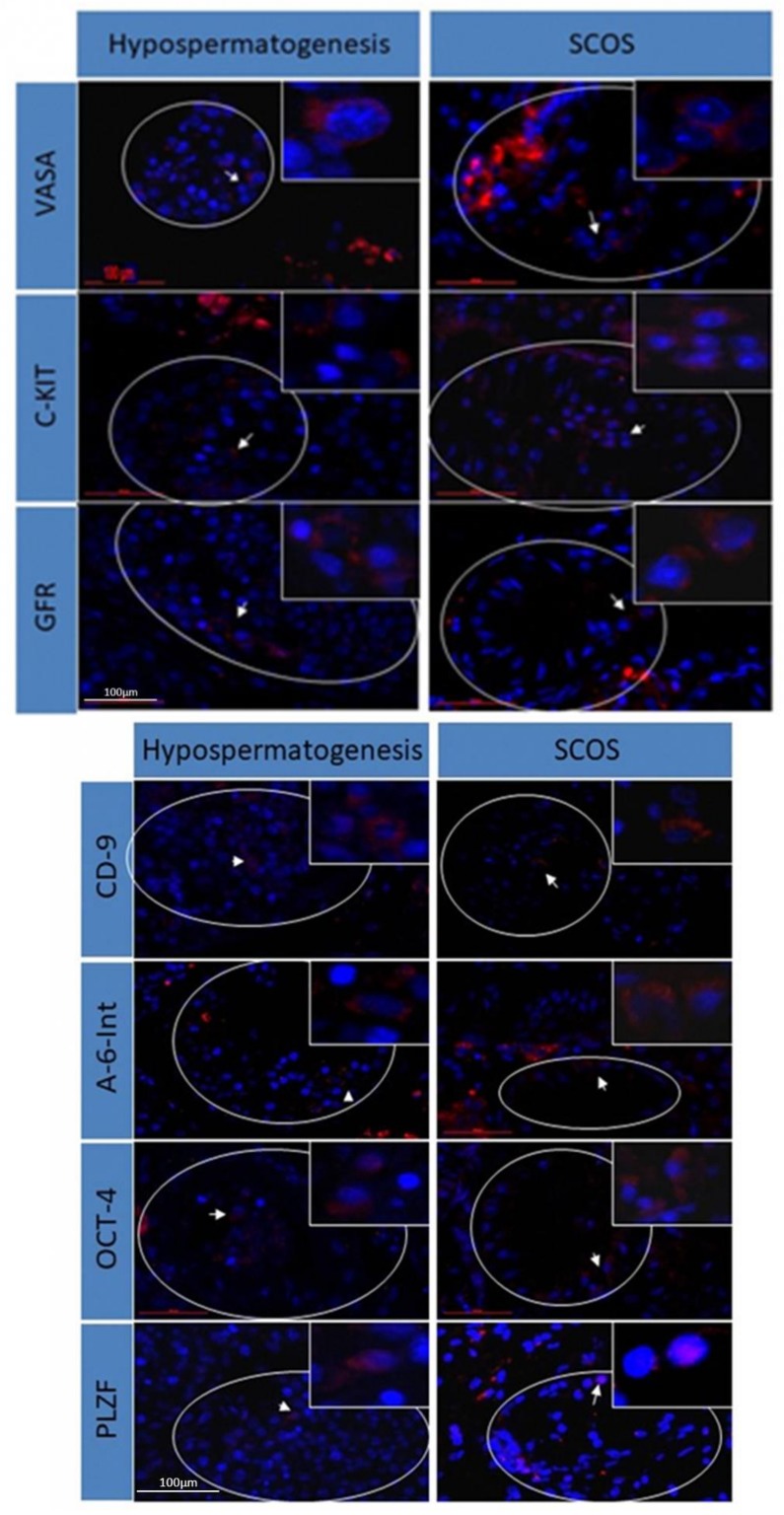
Immunofluorescence staining in hypospermatogenesis and SCOS testicular biopsies for the presence of premeiotic markers. Testicular biopsies with hypospermatogenesis and SCOS histology were examined for the presence of premeiotic cells by immunofluorescence staining using specific primary antibodies for each of the examined premeiotic markers: VASA, c-KIT, GFRa1, CD-9, a-6-Integrin, OCT-4, and PLZF. Blue—cell nuclei stained with DAPI, red—specific marker staining. Scale bar: 100 μm.

Biopsies from patients with hypospermatogenesis and SCOS were immunofluorescence-stained for the premeiotic markers VASA, c-KIT, GFR*a1*, CD-9, a-6-Integrin, OCT4, and PLZF (Figure 1). These markers were positively stained in biopsies of hypospermatogenesis (Hypo) patients that contained individual sperm (according to the In vitro Fertilization Unit (IVF) lab and histopathology) (*n* = 3) and patients with SCOS (according to biopsy histopathology) who did not have any sperm (according to the IVF lab) (*n* = 7).

The premeiotic markers were distinctly present/stained in the same group of patients and between the different groups. In the Hypo group, the range was from 1/3 to 3/3. In the SCOS group, the range was from 1/7 to 6/7.

### 2.3. Immunofluorescence Staining and RNA Expression of Premeiotic, Meiotic, and Postmeiotic Markers of Cells Isolated from Human Testicular Biopsies of Patients with Hypospermatogenesis and SCOS

**Figure 2:**
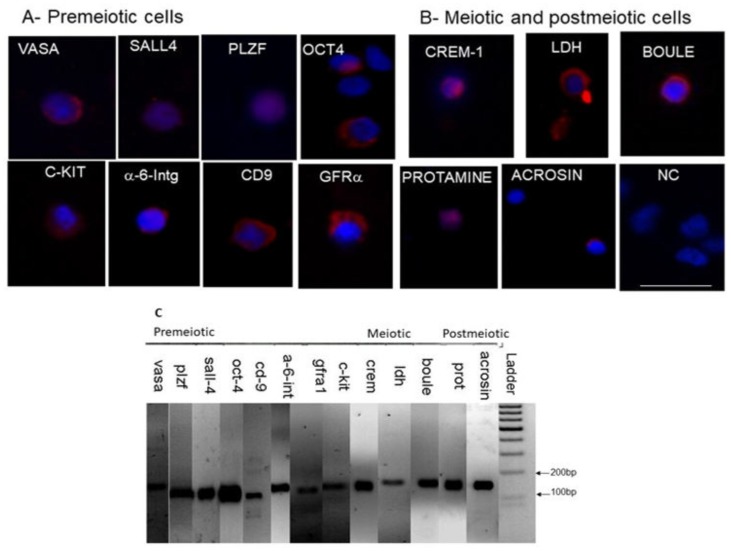
Immunofluorescence staining and RNA expression of spermatogenesis markers in cells isolated from biopsies of SCOS patients before and/or after culture. Enzymatically isolated cells from testicular biopsies of SCOS patients were examined by immunofluorescence (IF) staining for different markers of: (**A**) premeiotic stage (VASA, SALL4, PLZF, OCT4, c-KIT, α-6-Integrin, CD-9, and GFRa1); (**B**) meiotic stage (CREM-1, LDH, and BOULE) and postmeiotic stage (protamine and acrosin) using specific primary antibodies for each examined marker. Negative control (NC)—IF staining was performed without primary antibodies. Blue—cell nuclei stained with DAPI; red—specific marker staining. RT-PCR analysis was used to examine RNA expression of the spermatogenesis markers using specific primers for each marker (**C**). Negative control for PCR analyses was ultra-pure water instead of the cDNA. The sizes of the PCR products were 100bp–200bp. Scale bar: 100 μm.

Isolated cells from biopsies of patients with hypospermatogenesis or biopsies without sperm from patients with a SCOS diagnosis were examined by immunofluorescence staining (Figure 2A,B) or by PCR analysis (Figure 2C) for the presence or expression of premeiotic, meiotic, and postmeiotic stages.

Our results show biopsies without sperm-stained (IF) and/or expressed (RNA) premeiotic markers (two to seven markers/biopsy), meiotic markers (of nine biopsies, CREM and LDH were detected in five, and BOULE in three) and postmeiotic markers (protamine was detected in six and acrosin in three biopsies) (Table 2).

### 2.4. Expression of Spermatogenic Markers in Isolated Cells from Testicular Biopsies of SCOS Patients after In Vitro Culture

In order to evaluate the possible development of spermatogenesis in vitro in testicular biopsies without the sperm of SCOS patients, we isolated cells from these biopsies without sperm (*n* = 6) and cultured them in vitro in MCS for 3–7 weeks. We arrested the culture growth and development according to the quality of the developed cells/colonies in the culture based on cell morphology evaluated under the microscope. We compared the development/presence of premeiotic (OCT4, PLZF, VASA, GFRa1, CD9, a-6-Integrin, SALL4, and c-KIT), meiotic (CREM, LDH, and BOULE), and postmeiotic (protamine and acrosin) cells before and after culture by IF staining (representative of positive staining shown in Figure 2A,B) and/or by PCR analysis (representative of positive expression shown in Figure 2C).

**Figure 3:**
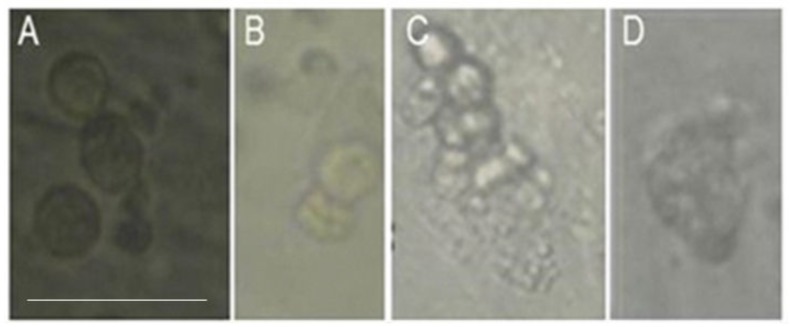
Morphology of developed cells/colonies from isolated cells from testicular biopsies of SCOS patients in-vitro. Germ cells from testicular biopsies without sperm of SCOS patients could form single cells and/or small, medium, and large colonies in MCS culture. (**A**)—single cells, (**A**,**B**)—pair cells, (**C**)—small colony (contained more than 10 cells and less than 50 cells), and (**D**)—medium colony (contained between 50 and 150 cells). Inverted microscope ×40. Scale bar: 100 μm.

During in vitro culture of the isolated cells from the biopsies, they were developed to single/pair or a-line cells (C; Cells) as seen in Figure 3A,B, or to small (S; around 50 cells) as seen in Figure 3C and medium colonies (M; around 100 cells) as seen in Figure 3D.

Our results show some biopsies (biopsies 5 and #6) that contained/expressed premeiotic markers before culture did not show their presence, and did not develop meiotic and postmeiotic cells after in vitro culture in the 3D system for 7 and 4 weeks, respectively (Table 3). Biopsy 9 that showed the presence/expression of premeiotic, meiotic, and postmeiotic cells before in vitro culture maintained some of the premeiotic cells (PLZF and VASA) and the meiotic marker BOULE after 3 weeks in vitro (Table 3). On the other hand, isolated cells from biopsy 7L that showed the presence/expression of some premeiotic, meiotic, and postmeiotic cells before in vitro culture maintained the presence of some premeiotic markers, and induced the expression of the meiotic markers LDH and BOULE and the postmeiotic marker acrosin after 6 weeks in vitro (Table 3). Isolated cells from biopsy 7R that showed the presence/expression of some premeiotic, meiotic, and postmeiotic cells before in vitro culture induced the presence of PLZF cells and the expression of c-KIT, maintained the expression of the meiotic marker CREM, and induced expression of the postmeiotic marker protamine after 6 weeks of in vitro culture (Table 3).

In addition, isolated cells from biopsy 4 that showed the presence/expression of some premeiotic, meiotic, and postmeiotic cells before in vitro culture maintained some premeiotic cells and induced GFRa cells and meiotic/postmeiotic acrosin-positive cells after 7 weeks in-vitro (Table 3).

## 3. Discussion

In the present study, we demonstrated for the first time the presence of meiotic and postmeiotic cells in some testicular biopsies without sperm from SCOS patients (according to an IVF search and histopathology). We also showed that testicular biopsies without sperm from different SCOS patients contain different subpopulations of premeiotic cells. These results may suggest that SCOS patients are a heterogeneous group, and that a diagnosis based on biopsy histopathology should be reconsidered. On the other hand, we cannot exclude the possibility of presence tubules at different stages of spermatogenesis (Sertoli cells only and incomplete maturation arrest) in the biopsy, however the part of the biopsy that we used for our cultures was different in its tubule composition from the part used in the histopathology examinations, which may lead to different results.

Even though we had very few cases (biopsies without sperm from SCOS patients) that were used to isolate the cells to be cultured in-vitro using the 3D system, our study shows for the first time the possibility of inducing the development of meiotic and postmeiotic cells in-vitro from those biopsies. These results may suggest that the premeiotic cells found/present in the testicular biopsies without sperm in SCOS patients are biologically active, with the potential to develop meiotic and postmeiotic cells. It is possible to suggest that the inability of those cells to complete spermatogenesis in vivo could be related to impairment in the testicular microenvironment and the functionality of Sertoli cells. The results of the present study are supported by our recent study, where we demonstrated the development of meiotic and postmeiotic cells including sperm-like cells in-vitro (in 3D MCS) from isolated cells from testicular biopsies of prepubertal male cancer patients before aggressive chemotherapy treatment [40]. We were also able to induce the development of sperm-like cells from normal immature mice in a 3D soft agar culture system [38] and from busulfan-treated immature mice in 3D MCS [41]. The results from the present study are encouraging, since they provide a proof of concept that some SCOS patients may have biologically active spermatogonial cells in their testicular biopsies with the ability to develop meiotic and postmeiotic stages in-vitro. From the examined biopsies we were unable to identify the development of sperm in the cultures.

These preliminary results show that our 3D system needs to be improved/optimized in order to find the optimal conditions to induce complete spermatogenesis in-vitro from SCOS patients, including the development of sperm. Some of the limitations to be overcome include the induction of greater proliferation of the spermatogonial cells in-vitro (since we started with a very low number of spermatogonial cells and the induction of proliferation may lead to the development of more differentiating cells including meiotic and postmeiotic cells), and also finding the conditions/factors to induce meiotic, postmeiotic stages and the generation of a mature/fertile sperm. The development of fertile sperm was demonstrated using organ cultures of testes from normal immature mice [36], but not yet from human. In addition, using germ cell transplantation technology and testicular grafting led to the development of fertile sperm [42,43]. These technologies have not yet been examined in humans. However, live offspring were reported from sperm generated from the testicular grafts of immature mice [44] and fertilization-competent sperm from testicular xenografts of immature rhesus monkeys into mice [45]. Unfortunately, experiments involving the xenografting of human prepubertal cryopreserved testicular tissue into immunodeficient mice have not yet demonstrated the development of spermatid differentiation [46,47,48,49].

In summary, we have shown that some biopsies without sperm from SCOS patients may contain premeiotic (biologically active), meiotic, and even postmeiotic cells. In this regard, we suggest extending the histopathology diagnosis by adding immunostaining for these types of cells in order to provide more accurate characterization for testicular biopsies with SCOS.

## 4. Materials and Methods

### 4.1. Human Testis Material

Local institutional ethical committees (Soroka University Medical Center Committee) approved the study (No. 4538; 07/07/2016). All patients signed informed consent documentation to use their testicular biopsy for research. Testicular specimens without sperm (*n* = 9) were obtained from seven azoospermic patients who were referred to the assisted reproductive technique in the IVF program due to absence of sperm in their ejaculate after centrifugation and a meticulous search of sperm cells. The specimens were obtained by testicular sperm extraction (TESE; *n* = 9 biopsies). The age of the patients ranged from 23–50 years. The TESE specimens were histologically identified as SCOS. Specimens were divided for histological evaluation and/or for RNA extraction, while others were also used for in vitro culture. None of the patients underwent surgery or chemotherapy/radiotherapy and their BMIs were in the range 20.98–29.04.

All patients were genetically evaluated including karyotyping and testing of whether they were normal for Y microdeletion. None were found to have Y microdeletions.

### 4.2. Testicular Cell Isolation and Culture

After overnight incubation in sperm wash media containing 30% human serum albumin at room temperature (RT), TESE specimens were re-searched for sperm and then collected in the same media and transferred to our lab. For cell isolation, biopsies were cut into small pieces (~2 mm) and subjected to enzymatic digestion as described in our previous study [41]. Cells were cultured (2 × 10^4^ cells/well/500 μL) in minimum essential media (MEM) as described in our previous study [41] and incubated for two nights in 24-well plates at 37 °C, 5% CO_2_. The nonadherent cells were collected and cultured (2 × 10^4^ cells/well/500 μL) in methylcellulose (R&D, Minneapolis, MN, USA) (42%) (as a 3D culture system), which contained 33% StemPro-34 medium (Gibco, Carlsbad, CA, USA) enriched with different factors and reagents as described previously [41]. Cells were cultured for 3–7 weeks. Every 1–2 weeks (according to cell growth and morphology), we added 50 μL/well of fresh concentrated (×10) enriched StemPro-34 medium to the culture.

Immunofluorescence staining: Immunofluorescence staining (IF) was performed using markers known to be specific for the different stages of spermatogenesis according to our previous studies [41].

Testicular tissues and cell staining: Testicular biopsies were fixed in 4% paraformaldehyde (Sigma) and paraffin-embedded. IF staining was performed using markers known to be specific for the different stages of spermatogenesis according to our previous studies [41].

Slides were examined for staining using a Nikon Eclipse 50i microscope (Tokyo, Japan).

Gene expression in examined cells: This was performed using markers known to be specific for the different stages of spermatogenesis according to our previous studies [41].

Evaluation of hormone levels: The hormone levels of FSH, LH, prolactin (Prolac), testosterone (T), and thyroid stimulating hormone (TSH) were examined in the blood of patients by radioimmunoassay.

## Figures and Tables

**Table 1:** Hormone levels in blood of SCOS patients. The levels of follicle stimulating hormone (FSH), luteinizing hormone (LH), prolactin (Prolac), and testosterone (T) were examined in the blood of SCOS patients without sperm by radioimmunoassay.

Hormone (HR) Pt#	FSH (0.7–11.0 mlU/mL)	LH (0.8–8.0 mlU/mL)	ProL (1–18 ng/mL)	Test. (2.45–12.0ng/mL)
1	19.6	4.8	6	1.96
3	22.5	15.8	13	4.3
4	14.5	31.9	7	4.4
5	14.4	35.9	17	4.3
6	16.3	7.4	41	1.7
7	8.9	5.4	5	5.52
9	30.9	8.9	35	5.77

**Table 2:** Presence/expression of spermatogenic markers in isolated cells from testicular biopsies without sperm of SCOS patients before in vitro culture: Isolated cells from biopsies without the sperm (*n* = 9) of SCOS patients were examined for premeiotic markers (OCT4, PLZF, VASA, GFRa1, CD9, α-6-Integrin, SALL4, and c-KIT) by immunofluorescence staining (IF) or by PCR analysis (R) using specific primary antibodies or primers (respectively) for each marker. The results (+) or (−) indicate the presence or absence (respectively) of the examined makers. Empty rectangles—indicate not examined. L—a biopsy from the left testis; R—a biopsy from the right testis.

Biopsy#	Pre-Meiotic Markers																Meiotic Markers						Post-Meiotic			
#	OCT4		PLZF		VASA		GFR		CD9		α-6-INT		SALL4		cKIT		CREM		LDH		BOULE		PROTAMINE		ACROSIN	
	IF	R	IF	R	IF	R	IF	R	IF	R	IF	R	IF	R	IF	R	IF	R	IF	R	IF	R	IF	R	IF	R
1L		−		−								−				+		−		−				−		−
1R		+								−				+		−		−		−				−		−
3	−	+	+	+	−	+	−	−	−	−	−		−	+	−	+	−	+	−	+	+	−	−	+	−	+
4	+	+	−		−		+	+	+	+	−		+	+	+	−	−	+	−	−	+		−	+	−	−
5	+		−		−		+	+	−		+		−		−		−		−		−		−		−	
6	+		+		+		−	−	+		+		+		+		+		+		−		+		−	
7L	+	+	−	+	+	+	+	+	+	−	+	+	−	+	−	−	−	+	−	+	−	−	−	+	−	+
7R	+	+	−	+	+	+	−	−	−	+	−	−	+	+	−	+	+	+	+	−	−	−	−	+	−	+
9	−	+	−	+	−	+	−	−	−	−	−	+	−	−	+	+	−	−	−	+	−	+	−	+	−	−

**Table 3:** Expression of spermatogenic markers in developed cells/colonies in MCS isolated from testicular biopsies without sperm of SCOS patients: Cells isolated from testicular biopsies without sperm (*n* = 6) from SCOS patients were cultured for a number of weeks (w) in MCS in-vitro. Cells before and after culture in MCS were examined for the presence of spermatogenic cells by immunofluorescence staining (IF) or for expression markers (RNA; R) by PCR analysis using specific antibodies or primers (respectively) for each spermatogenic stage: premeiotic (OCT4, PLZF, VASA, GFRa1, CD9, α-6-Integrin, SALL4, and c-KIT), meiotic (CREM, LDH, and BOULE), and postmeiotic (protamine and acrosin). BC—before culture, AC—after culture, W—number of weeks in culture, C—cells, S—small colonies, M—medium colonies. Empty rectangle—indicates not examined. †—indicates markers that were not expressed before in vitro culture and positively expressed after in vitro culture in MCS. *—indicates markers that were expressed before in vitro culture and continued to be positively expressed after in vitro culture in MCS. (−)—indicates negative expression, (+)—indicates positive expression. L—a biopsy from the left testis; R—a biopsy from the right testis.

Biopsy#	BC/AC	weeks	Pre-Meiotic Markers																Meiotic Markers							Post-Meiotic				Colonies
			OCT4		PLZF		VASA		GFR		CD9		α-6-INT		SALL4		cKIT		CREM			LDH		BOULE		PROTAMINE		ACROSIN		
			IF	R	IF	R	IF	R	IF	R	IF	R	IF	R	IF	IF	R	IF	R	IF	R	IF	R	IF	R	IF	R	IF	
4	BC		+	+*	−		−		+	−^†^	+	+	−		+	+*	+	−	−	+*	−		−	+		−	+*	−	−^†^	
	AC	7w	−	+*	−	+	−	+	+	+^†^	−	−	−	+	−	+*	−	−	+	+*	−		−	−	-	−	+*	−	+^†^	C
5	BC		+		−		−		+		−		+		−		−		−		−			−		−		−		
	AC	7w	−		−		−		−		−		−		−		−		−		−			−		−		−		C, S, M
6	BC		+		+		+		−		+		+		+		+		+		+			−		+		−		
	AC	4w	−		−		−		−		−		−		−		−		−		−			−		−		−		S
7L	BC		+*	+	−	+	+*	+	+*	+	+	−	+*	+	−	+	−	−	−	+	−^†^		+	−^†^	−	−	+	−^†^	+	
	AC	6w	+*		−		+*		+*		−		+*		−		−		−		+^†^			+^†^		−		+^†^		S, M
7R	BC		+	+	−^†^	+	+	+	−	+	−	+	−	−	+	+	−^†^	+	+*	+	+		−	−	−	−^†^	+	−	+	
	AC	6w	−		+^†^		−		−		−		−		−		+^†^		+*		−			−		+^†^		−		C, S
9	BC		−	+	−	+*	−	+*	−	+	−	−	-	+	−	−	+	+	−	−	−		+	−	+*	−	+	−	-	
	AC	3w		−		+*		+*		−		−		−		−		−		−			-		+*		−		-	S, M
